# Predictors of Shift Work Sleep Disorder Among Nurses During the COVID-19 Pandemic: A Multicenter Cross-Sectional Study

**DOI:** 10.3389/fpubh.2021.785518

**Published:** 2021-12-02

**Authors:** Yuxin Li, Xiaoyan Lv, Rong Li, Yongchao Wang, Xiangyun Guan, Li Li, Junli Li, Fuzhong Xue, Xiaokang Ji, Yingjuan Cao

**Affiliations:** ^1^School of Nursing and Rehabilitation, Cheeloo College of Medicine, Shandong University, Jinan, China; ^2^Department of Nursing, Qilu Hospital, Cheeloo College of Medicine, Shandong University, Jinan, China; ^3^Nursing Theory and Practice Innovation Research Center, Cheeloo College of Medicine, Shandong University, Jinan, China; ^4^Department of Biostatistics, School of Public Health, Cheeloo College of Medicine, Shandong University, Jinan, China; ^5^Institute for Medical Dataology, Shandong University, Jinan, China

**Keywords:** China, cross-sectional studies, nurses, personnel staffing and scheduling, shift work sleep disorder

## Abstract

**Background:** Nurses have a high incidence of shift work sleep disorder, which places their health and patient safety in danger. Thus, exploring the factors associated with shift work sleep disorder in nurses is of great significance in improving their sleep health, nursing personnel staffing, and scheduling during the COVID-19 pandemic.

**Objectives:** The purpose of this study was to investigate the incidence of shift work sleep disorder during the COVID-19 pandemic and explore the factors associated with shift work sleep disorder in Chinese nurses.

**Methods:** This was a multicenter cross-sectional study using an online survey. Stratified cluster sampling was used to include 4,275 nurses from 14 hospitals in Shandong, China from December 2020 to June 2021. Stepwise multivariate logistic regression analysis and random forest were used to identify the factors associated with shift work sleep disorder.

**Results:** The prevalence of shift work sleep disorder in the sampled shift nurses was 48.5% during the COVID-19 pandemic. Physical fatigue, psychological stress, shift work more than 6 months per year, busyness during night shift, working more than 40 h per week, working more than four night shifts per month, sleeping more than 8 h before night shift, using sleep medication, irregular meals, and high-intensity physical activity were associated with increased odds of shift work sleep disorder. Good social support, good work-family balance, napping two or three times per week, resting more than one day after shifts, intervals of 8 days or more between shifts, and taking turns to rest during the night shift were associated with decreased odds of shift work sleep disorder.

**Conclusions:** Shift work sleep disorder may be associated with scheduling strategies and personal behavior during the COVID-19 pandemic. To reduce the incidence of shift work sleep disorders in nurses, nursing managers should increase night shift staffing, extend rest days after shift, increase night shift spacing, and reduce overtime, and nurses need to seek more family and social support and control their sleep schedules and diet.

## Introduction

The National Sleep Foundation defines shift work as a work that takes place outside the traditional daily schedule of 09:00–17:00 (1). Shift work is very common in the healthcare sector, in which nurses are usually involved. A previous longitudinal study showed that shift work schedules had no effect on lifestyle (2). However, shift work interferes with biological rhythms, and can lead to a wide variety of health problems, with sleep problems being the most common health problem (3). Thus, shift work sleep disorder (SWSD) is considered a chronic condition that is directly related to work schedule (4). Previous studies have shown that nurses are taking more frequent shifts, with heavier workloads and a sharp increase in fatigue and mental stress during the COVID-19 pandemic (5). Large numbers of patients, long working hours and the definition of new roles for healthcare workers in the context of the COVID-19 pandemic have led to insufficient breaks and sleep, and reduced job satisfaction (6). However, previous studies have lacked reports on the prevalence of SWSD for nurses during the COVID-19 pandemic. Therefore, nursing managers and nurses themselves should improve their awareness of the incidence and harm of SWSD during the COVID-19 pandemic, and pay more attention to the possible factors associated with SWSD in the physical, psychological, environmental, occupational, social, and behavioral aspects, so as to improve professional identity and happiness and ensure the health and safety of nurses on duty.

In 2014, the third edition of ICSD (ICSD-3) defined SWSD as a circadian rhythm disorder and updated the ICSD-2 criteria for this condition (7, 8). Major changes from ICSD-2 include linking insomnia/sleepiness to a reduction in the total sleep time associated with work schedules. In addition, the minimum duration of symptoms increased from 1 month to at least 3 months (9). The incidence of SWSD among nurses is reported to be between 24.4 and 37.6% using ICSD-2 criteria, accounting for a quarter of the total number of nurses (10, 11). In a Norwegian study using ICSD-3 criteria, 45.6% of nurses reported previously experiencing SWSD during 2 years of follow-up (4). However, a recent study in Finland reported that using ICSD-3 criteria led to a lower incidence of SWSD diagnosis compared to using ICSD-2 criteria (12). Thus far, few studies have investigated the prevalence of SWSD according to the latest criteria, and its prevalence according to country and occupation remains to be determined (13).

SWSD is associated with serious adverse consequences for individuals, employers, and society, and can damage health (1). In addition, there are many health problems that occur with SWSD, such as anxiety or depression (14), headaches (15), and impaired cognitive function (16). SWSD is associated with lower alertness, reduced attention, and increased likelihood of sleepiness-related accidents, all of which can reduce work performance, patient safety, and quality of life (13, 17–19). In addition, SWSD leads to loss of productivity and increased economic costs (20). The dissonance between endogenous circadian rhythms and sleep and work schedules of shift workers may be the mechanism responsible for SWSD (17). In addition, short rest periods (<11 h) between two shifts have also been reported to be associated with the presence of SWSD (21). Previous reviews have also pointed to the importance of investigating the association of shift work schedules and lifestyle factors with SWSD and exploring preventive measures for SWSD (1, 20). Jang proposed interventions to manage SWSD, including adjusting the shift work schedules and working hours, providing shift workers with adequate sleep health education, and providing the opportunities to take naps and breaks during night shifts (20). However, specifics of the factors and interventions, including periods between two shifts, staffing, frequency and duration of naps, sleep duration before and after night shifts, and dietary adjustments, remain unclear (13, 17).

Therefore, in this study we first aimed to measure the incidence of SWSD in nurses based on the latest diagnostic criteria. Second, we aimed to explore the comprehensive factors of SWSD in shift nurses from physical, psychological, environmental, occupational, social, and behavioral perspectives based on the Integrated Framework for Population Health Risk Management (22) to fill the gaps in extant research. Furthermore, random forest was used to rank the importance of the factors. Currently, nursing management emphasizes on making full use of strengths and avoiding weaknesses, and our study intended to find approaches that will aid in scientifically and effectively organizing shift schedules for nurses to reduce the incidence of SWSD, promote health, and improve the quality of life of shift nurses.

## Materials and Methods

### Design, Setting, and Participants

In this multicenter cross-sectional study, we used data from the first survey of Chinese nurses conducted between December 2020 to June 2021 from the Nurses' Health Cohort Study of Shandong. The cohort study mentioned above is an ongoing prospective cohort study of Chinese nurses conducted in Shandong province, China (registration number: ChiCTR2100043202). The study adopted a multistage sampling method for a more extensive sample coverage. The steps are as follows: (a) Shandong was categorized into eastern, western, southern, northern and central regions; (b) hospitals were selected by convenience sampling (at least one secondary and one tertiary hospital were selected from each region); and (c) participants were selected through cluster sampling.

Registered nurses with nurse qualification certificates who worked in shifts, and agreed to participate in this study were included in the present study. Retired nurses, refresher nurses, and student nurses; nurses who had been working for a duration of <6 months; and nurses who were on leave during the investigation were excluded from the present study.

### Measures

The questionnaire consisted of the following four parts: demographic and lifestyle variables; SWSD assessment; shift work characteristics; standardized questionnaires.

#### Demographic and Lifestyle Variables

The basic demographic variables included sex, age, marital status, children, educational background, department, hospital level, professional title, and income. The body mass index was calculated as body weight (kg) divided by the squared height (m^2^), and nurses were categorized on the basis of their body mass index as underweight (≤18.4), normal weight (18.5–23.9), and overweight (≥24) (23). Lifestyle variables included drinking caffeinated beverages at work (yes/no), sleep duration before and after the night shift, use of sleep-promoting drugs (yes/no), changes in diet during shift work, and the frequency and duration of naps.

#### Shift Work Characteristics

Shift work characteristics included shift schedule (two shifts/three shifts/other), direction of shift rotation (clockwise/counterclockwise), intervals between night shifts, number of years working in shift work, number of hours of working/overtime per week, number of months of shift work per year, number of night shifts per month, busyness during night shift (yes/no), psychological burden during shift work (yes/no), and physical discomfort during shift work (yes/no).

#### SWSD Assessment

SWSD was assessed with the following three questions: (a) Do you have a work schedule that sometimes overlaps with the time you usually sleep? (b) If yes, does this cause insomnia and/or excessive sleepiness due to reduced amount of sleep? (c) If yes, has this lasted for at least three months? Participants who responded “yes” to all three questions were classified as having SWSD (4).

#### Work-Family Balance Scale

The work-family balance was assessed using a 14-item work-family balance scale, which is widely used to measure the work-family inter-relation based on four domains, namely, work-family conflict, family-work conflict, work-family enrichment, and family-work enrichment (24). This scale was adopted and modified by Chinese researchers and had good reliability and validity (25). Each item is rated on a 5-point Likert scale from 1 (definitely disagree) to 5 (definitely agree). Based on the total score, work-family balance was recategorized as high (>42) and low ( ≤ 42). In the present study, the Cronbach's alpha coefficient of the scale was 0.811.

#### Perceived Social Support Scale

Social support was assessed using the 12-item perceived social support scale, which is a reliable instrument for measuring perceived support from family, friends, and others (26). Responses for each item range from 1 (definitely disagree) to 7 (definitely agree). Based on the total score, shift nurses were categorized as having low (12–36), medium (37–60), and high (61–84) levels of perceived social support. The Chinese version has shown good psychometric properties with a Cronbach's alpha coefficient of 0.91 and a test-retest reliability of 0.85 (27). The Cronbach's alpha coefficient of the scale in this study was 0.980.

#### Fatigue Scale

The fatigue scale was used to measure the severity of fatigue (28). This 14-item questionnaire includes two dimensions: physical fatigue and mental fatigue. Nurses were categorized into different levels of fatigue on the basis of their total score on the scale: low (0–4), medium (5–9), and high (10–14). The Chinese version of the scale has been widely used with had good reliability and validity (29). The Cronbach's alpha coefficient of the scale in this study was 0.803.

#### Perceived Stress Scale

The 10-item perceived stress scale was used to assess nurses' perceptions of stress (30). The Chinese version of the perceived pressure scale adopted in this study has been verified to have good reliability and validity (31). Each item is measured on a 5-point Likert scale from 0 (never) to 4 (always), and the perceived stress level was divided into three categories: low (0–13), medium (14–27), and high (28–40). The Cronbach's alpha coefficient of the scale in this study was 0.741.

#### Circadian Type Inventory

A 11-item circadian type inventory was previously developed to measure circadian rhythms (32). In the current study, we used the Chinese version of the scale to assess circadian flexibility and languidity (33). The two subscales used in this scale were divided into low, medium, and high levels based on the one-third and two-third cutoff points of the theoretical subscale score. A higher level on the flexibility subscale indicated greater adaptability to shifts, while a higher level on languidity indicated greater difficulty in overcoming sleepiness. The Cronbach's alpha coefficient of the scale in this study was 0.838.

#### International Physical Activity Questionnaire

The international physical activity questionnaire has been widely used to study the physical activity level (34). The questionnaire includes five parts: occupation, housework, transportation, leisure, and sitting. We used the Chinese version of the questionnaire, which has good reliability and validity (35). We divided the physical activity into three levels by calculating the total metabolic equivalent: low, medium, and high.

### Data Collection

Baseline information was collected through the Wenjuanxing network platform between December 2020 to June 2021. The survey included eight electronic questionnaires, each of which would take about 10–15 min to complete. Nurses were sent these questionnaires via WeChat (a widely used instant messaging app used in China) in batches to avoid impatience. Subjects who did not participate in the survey within 1 week of sending the questionnaires received a WeChat reminder to complete the survey once per week until the questionnaires were completed. Liaisons were set in every hospital involved in this study, functioning as regulators for questionnaire completion and communicators with the researchers when facing unsolved problems. Only professionals who signed nondisclosure agreements had direct access to the data. To ensure data quality, we adopted the required questions and data logic control design for the electronic questionnaires.

### Ethical Considerations

This study was carried out in accordance with the International Guidelines of Good Epidemiology Practice and the Declaration of Helsinki principles. The nurses' participation was voluntary, and written informed consents were obtained from all the respondents. The study was approved by the Ethics Committee of Scientific Research of Hospital with the registration number (KYLL-202011-085).

### Data Analysis

All the variables in this study, including one dependent variable and 43 independent variables, were categorical variables. Descriptive statistics included frequency and percentage for them. The distribution of characteristics between shift nurses with and without SWSD was assessed using the chi-squared test or Fisher's exact test for categorical variables. Bonferroni correction was applied for correcting the multiple test and *p* < 0.0012 was considered statistically significant for the univariate analysis. Statistically significant variables in the univariate analyses were all considered for inclusion in a forward and backward stepwise multivariate logistic regression analysis to identify the main factors associated with SWSD and to estimate the adjusted odds ratios (ORs) and 95% confidence intervals (CIs). The final logistic regression model with Akaike Information Criterion (AIC) minimization was used, and variables in the final model with *p* < 0.05 denoted statistically significant differences. Logistic regression was used because this classic classifier has been widely used in disease prediction and it is easy to interpret the results.

In order to more accurately explore the importance of factors, we adopted a machine learning method, random forest. The random forest is a tree-based ensemble classification algorithm, which has been widely used to build disease prediction models with small variable sets and high generalization ability. We use grid search for hyperparameter adjustment when training the model. The data was divided into 7:3 training data and test data. The training set was used to train and obtain the final model, and the test set was used to draw receiver-operating characteristic (ROC) curves to determine the prediction performance of the model. The random forest was implemented using Python 3.7.4 (Python Software Foundation, USA) with Scikit-learn (Packt Publishing, UK). Other statistical analyses were carried out using R software version 4.0.5 (R Development Core Team, Vienna, Austria).

## Results

### Respondent Characteristics and SWSD Prevalence

The participants (*n* = 4,655) of the present study were recruited from nurses participating in shift work in 14 hospitals across six cities in Shandong, China. Of these, nurses who did not complete the questionnaire or those whose questionnaires contained logical inconsistencies were eliminated, leading to the exclusion of 8.17% of respondents, leaving 4,275 participants. The study participants ranged in age from 21 to 55 years and 93.2% (3,985) were women. The average age of the respondents was 31.19 ± 5.48 years, and the average working duration was 8.62 ± 5.16 years. In this study, the prevalence of SWSD among participating nurses was as high as 48.5%. Table 1 shows all the respondents' features and univariate analysis of SWSD in detail.

**Table 1:** Respondent characteristics and univariate analysis of factors related to shift work sleep disorder in shift nurses.

		**Shift work sleep disorder**			
**Variables**	***N* (%)**	**No, *N* (%)**	**Yes, *N* (%)**	**χ^2^ value**	***p* value^†^**
**Total**	4,275 (100.0)	2,202 (51.5)	2,073 (48.5)		
**I. Physical and psychological factors**					
**Gender**					
Male	290 (6.8)	136 (6.2)	154 (7.4)	2.650	0.104
Female	3,985 (93.2)	2,066 (93.8)	1,919 (92.6)		
**Age**					
≤ 25 years	678 (15.9)	414 (18.8)	264 (12.7)	43.898	**<0.001** ^*^
26–30 years	1,124 (26.3)	603 (27.4)	521 (25.1)		
31–35 years	1,711 (40.0)	843 (38.3)	868 (41.9)		
36–40 years	609 (14.2)	276 (12.5)	333 (16.1)		
>40 years	153 (3.6)	66 (3.0)	87 (4.2)		
**Body mass index**					
Underweight	461 (10.8)	234 (10.6)	227 (11.0)	2.117	0.347
Normal weight	2,651 (62.0)	1,388 (63.0)	1,263 (60.9)		
Overweight	1,163 (27.2)	580 (26.3)	583 (28.1)		
**Circadian rhythms^‡^**					
**Flexibility**					
Low	433 (10.1)	212 (9.6)	221 (10.7)	1.283	0.527
Moderate	2,673 (62.5)	1,387 (63.0)	1,286 (62.0)		
High	1,169 (27.3)	603 (27.4)	566 (27.3)		
**Languidity**					
Low	530 (12.4)	258 (11.7)	272 (13.1)	2.243	0.326
Moderate	3,000 (70.2)	1,564 (71.0)	1,436 (69.3)		
High	745 (17.4)	380 (17.3)	365 (17.6)		
**Fatigue**					
Low	903 (21.1)	677 (30.7)	226 (10.9)	461.458	**<0.001** ^*^
Moderate	1,335 (31.2)	809 (36.7)	526 (25.4)		
High	2,037 (47.6)	716 (32.5)	1,321 (63.7)		
**Physical discomfort during night shift**					
No	1,427 (33.4)	1,037 (47.1)	390 (18.8)	384.021	**<0.001** ^*^
Yes	2,848 (66.6)	1,165 (52.9)	1,683 (81.2)		
**Psychological stress before night shift**					
No	466 (10.9)	364 (16.5)	102 (4.9)	148.191	**<0.001** ^*^
Yes	3,809 (89.1)	1,838 (83.5)	1,971 (95.1)		
**Psychological stress during night shift**					
No	503 (11.8)	395 (17.9)	108 (5.2)	166.633	**<0.001** ^*^
Yes	3,772 (88.2)	1,807 (82.1)	1,965 (94.8)		
**Psychological stress after night shift**					
No	1,765 (41.3)	1,170 (53.1)	595 (28.7)	262.919	**<0.001** ^*^
Yes	2,510 (58.7)	1,032 (46.9)	1,478 (71.3)		
**Perceived stress**					
Low	541 (12.7)	390 (17.7)	151 (7.3)	193.251	**<0.001** ^*^
Moderate	3,579 (83.7)	1,794 (81.5)	1,785 (86.1)		
High	155 (3.6)	18 (0.8)	137 (6.6)		
**II. Environmental and occupational factors**					
**Hospital level** ^§^					
Secondary hospital	1,415 (33.1)	764 (34.7)	651 (31.4)	5.226	**0.022**
Tertiary hospital	2,860 (66.9)	1,438 (65.3)	1,422 (68.6)		
**Departments**					
Internal medicine	1,317 (30.8)	723 (32.8)	594 (28.7)	39.945	**<0.001** ^*^
Surgery	995 (23.3)	476 (21.6)	519 (25.0)		
Emergency	292 (6.8)	122 (5.5)	170 (8.2)		
Gynecology and obstetrics	312 (7.3)	160 (7.3)	152 (7.3)		
Pediatrics	390 (9.1)	215 (9.8)	175 (8.4)		
Operating room	282 (6.6)	164 (7.4)	118 (5.7)		
Intensive care unit	330 (7.7)	144 (6.5)	186 (9.0)		
Others	357 (8.4)	198 (9.0)	159 (7.7)		
**Professional title**					
Primary	2,878 (67.3)	1,573 (71.4)	1,305 (63.0)	35.250	**<0.001** ^*^
Medium	1,365 (31.9)	613 (27.8)	752 (36.3)		
Senior	32 (0.7)	16 (0.7)	16 (0.8)		
**Monthly income, Chinese Yuan**					
<3,000	346 (8.1)	198 (9.0)	148 (7.1)	27.034	**<0.001** ^*^
3,000–5,999	2,534 (59.3)	1,362 (61.9)	1,172 (56.5)		
6,000–8,999	1,155 (27.0)	537 (24.4)	618 (29.8)		
≥9,000	240 (5.6)	105 (4.8)	135 (6.5)		
**Work schedule**					
Two shifts	2,286 (53.5)	1,206 (54.8)	1,080 (52.1)	14.330	**0.001** ^*^
Three shifts	1,833 (42.9)	938 (42.6)	895 (43.2)		
Others	156 (3.6)	58 (2.6)	98 (4.7)		
**Direction of shift rotation**					
Clockwise	2,740 (64.1)	1,458 (66.2)	1,282 (61.8)	8.860	**0.003**
Counterclockwise	1,535 (35.9)	744 (33.8)	791 (38.2)		
**Shift work experience**					
0–5 years	1,371 (32.1)	795 (36.1)	576 (27.8)	65.905	**<0.001** ^*^
6–10 years	1,532 (35.8)	818 (37.1)	714 (34.4)		
11–15 years	999 (23.4)	433 (19.7)	566 (27.3)		
16–20 years	288 (6.7)	120 (5.4)	168 (8.1)		
>20 years	85 (2.0)	36 (1.6)	49 (2.4)		
**Months of shift work per year**					
1–3 months	209 (4.9)	147 (6.7)	62 (3.0)	73.364	**<0.001** ^*^
4–6 months	385 (9)	248 (11.3)	137 (6.6)		
7–9 months	432 (10.1)	242 (11.0)	190 (9.2)		
10–12 months	3,249 (76.0)	1,565 (71.1)	1,684 (81.2)		
**Night shifts per month**					
≤ 4	884 (20.7)	561 (25.5)	323 (15.6)	92.829	**<0.001** ^*^
5–9	2,963 (69.3)	1,486 (67.5)	1,477 (71.2)		
≥10	428 (10.0)	155 (7.0)	273 (13.2)		
**Days off after night shift**					
1 day	2,524 (59.0)	1,164 (52.9)	1,360 (65.6)	76.881	**<0.001** ^*^
1.5 days	538 (12.6)	297 (13.5)	241 (11.6)		
≥2 days	1,213 (28.4)	741 (33.7)	472 (22.8)		
**Interval between night shifts**					
≤ 4 days	1,388 (32.5)	635 (28.8)	753 (36.3)	38.403	**<0.001** ^*^
5–7 days	2,412 (56.4)	1,276 (57.9)	1,136 (54.8)		
≥8 days	475 (11.1)	291 (13.2)	184 (8.9)		
**Night shift staffing**					
1	1,248 (29.2)	643 (29.2)	605 (29.2)	23.532	**<0.001** ^*^
2 (take turns)	1,601 (37.5)	891 (40.5)	710 (34.2)		
2 (take no turns)	649 (15.2)	301 (13.7)	348 (16.8)		
≥3	777 (18.2)	367 (167)	410 (19.8)		
**Busyness during night shift**					
No	1,146 (26.8)	754 (34.2)	392 (18.9)	127.923	**<0.001** ^*^
Yes	3,129 (73.2)	1,448 (65.8)	1,681 (81.1)		
**Working** **>40 h/week**					
Never/rarely	772 (18.1)	550 (25.0)	222 (10.7)	253.197	**<0.001** ^*^
1 week/month	796 (18.6)	480 (21.8)	316 (15.2)		
2 weeks/month	713 (16.7)	367 (16.7)	346 (16.7)		
3 weeks/month	462 (10.8)	215 (9.8)	247 (11.9)		
4 weeks/month	1,532 (35.8)	590 (26.8)	942 (45.4)		
**Working during off-hours per month**					
≤ 1 day	3,173 (74.2)	1,746 (79.3)	1,427 (68.8)	62.278	**<0.001** ^*^
2 days	613 (14.3)	263 (11.9)	350 (16.9)		
≥3 days	489 (11.4)	193 (8.8)	296 (14.3)		
**III. Social and behavioral factors**					
**Education^¶^**					
Secondary vocational degree	697 (16.3)	347 (15.8)	350 (16.9)	6.034	**0.049**
Associate's degree	2,744 (64.2)	1,451 (65.9)	1,293 (62.4)		
Bachelor's degree	834 (19.5)	404 (18.3)	430 (20.7)		
**Marital status**					
Unmarried	1,272 (29.8)	720 (32.7)	552 (26.6)	22.235	**<0.001** ^*^
Married	2,926 (68.4)	1,452 (65.9)	1,474 (71.1)		
Others	77 (1.8)	30 (1.4)	47 (2.3)		
**Children**					
0	1,705 (39.9)	933 (42.4)	772 (37.2)	12.048	**0.002**
1	1,323 (30.9)	646 (29.3)	677 (32.7)		
≥2	1,247 (29.2)	623 (28.3)	624 (30.1)		
**Work-family balance**					
Low	358 (8.4)	62 (2.8)	296 (14.3)	182.864	**<0.001** ^*^
High	3,917 (91.6)	2,140 (97.2)	1,777 (85.7)		
**Social support**					
Low	726 (17.0)	347 (15.8)	379 (18.3)	78.836	**<0.001** ^*^
Moderate	1,452 (34.0)	633 (28.7)	819 (39.5)		
High	2,097 (49.1)	1,222(55.5)	875 (42.2)		
**Naps during the night shift**					
Never/rarely	3,209 (75.1)	1,634 (74.2)	1,575 (76.0)	7.247	0.123
≤ 1 h	125 (2.9)	66 (3.0)	59 (2.8)		
1–2 h	353 (8.3)	171 (7.8)	182 (8.8)		
2–3 h	355 (8.3)	200 (9.1)	155 (7.5)		
>3 h	233 (5.5)	131 (5.9)	102 (4.9)		
**Sleep duration before night shift**					
<3 h	2,842 (66.5)	1,364 (61.9)	1,478 (71.3)	53.094	**<0.001** ^*^
3–5 h	1,227 (28.7)	735 (33.4)	492 (23.7)		
6–8 h	136 (3.2)	75 (3.4)	61 (2.9)		
>8 h	70 (1.6)	28 (1.3)	42 (2.0)		
**Sleep duration after night shift**					
<3 h	1,375 (32.2)	590 (26.8)	785 (37.9)	65.071	**<0.001** ^*^
3–5 h	2,398 (56.1)	1,327 (60.3)	1,071 (51.7)		
6–8 h	389 (9.1)	231 (10.5)	158 (7.6)		
>8 h	113 (2.6)	54 (2.5)	59 (2.8)		
**Naps per week**					
Never/rarely	912 (21.3)	434 (19.7)	478 (23.1)	32.000	**<0.001** ^*^
≤ 1 day	1,022 (23.9)	484 (22.0)	538 (26.0)		
2–3 days	1,615 (37.8)	862 (39.1)	753 (36.3)		
4–5 days	509 (11.9)	283 (12.9)	226 (10.9)		
6–7 days	217 (5.1)	139 (6.3)	78 (3.8)		
**Using sleep medication before night shift**					
Often	152 (3.6)	30 (1.4)	122 (5.9)	268.113	**<0.001** ^*^
Sometimes	800 (18.7)	240 (10.9)	560 (27.0)		
Never/rarely	3,323 (77.7)	1,932 (87.7)	1,391 (67.1)		
**Using sleep medication after night shift**					
Often	149 (3.5)	21 (1.0)	128 (6.3)	274.766	**<0.001** ^*^
Sometimes	666 (15.6)	190 (8.6)	476 (23.0)		
Never/rarely	3,460 (80.9)	1,991 (90.4)	1,469 (70.9)		
**Food intake during shift work**					
More	515 (12.0)	197 (8.9)	318 (15.3)	151.815	**<0.001** ^*^
Normal	1,439 (33.7)	924 (42.0)	515 (24.8)		
Less	2,321 (54.3)	1,081 (49.1)	1,240 (59.8)		
**Time of meal during shift work**					
Early	746 (17.5)	330 (15.0)	416 (20.1)	151.621	**<0.001** ^*^
Regular	1,591 (37.2)	1,014 (46.0)	577 (27.8)		
Delayed	1,938 (45.3)	858 (39.0)	1,080 (52.1)		
**Drinking caffeinated beverage at work**					
No	2,995 (70.1)	1,603 (72.8)	1,392 (67.1)	16.240	**<0.001** ^*^
Yes	1,280 (29.9)	599 (27.2)	681 (32.9)		
**Water intake during work**					
≤ 500 mL	2,279 (53.3)	1,073 (48.7)	1,206 (58.2)	38.898	**<0.001** ^*^
501–1,000 mL	1,598 (37.4)	897 (40.7)	701 (33.8)		
1,001–1,500 mL	260 (6.1)	152 (6.9)	108 (5.2)		
>1,500 mL	138 (3.2)	80 (3.6)	58 (2.8)		
**Whole day water intake**					
≤ 500 mL	690 (16.1)	314 (14.3)	376 (18.1)	17.118	**0.001** ^*^
501–1,000 mL	2,008 (47.0)	1,026 (46.6)	982 (47.4)		
1,001–1,500 mL	956 (22.4)	531 (24.1)	425 (20.5)		
>1,500 mL	621 (14.5)	331 (15.0)	290 (14.0)		
**Physical activity**					
Low	1,397 (32.7)	791 (35.9)	606 (29.2)	40.527	**<0.001** ^*^
Moderate	878 (20.5)	484 (22.0)	394 (19.0)		
High	2,000 (46.8)	927 (42.1)	1,073 (51.8)		

*Bold value for p < 0.05*.

* *Statistically significant differences after application of Bonferroni correction (p < 0.0012)*.

† *p value for chi-squared test*.

‡ *Circadian rhythms include both circadian flexibility and languidity*.

§ *Hospital level management divides hospitals into primary, secondary, and tertiary hospitals*.

¶ *Secondary vocational degree: A 4-year senior high school for professional training; associate degree: A 3-year college course for professional training; bachelor's degree: A 4- or 5-year undergraduate training course*.

### Stepwise Multivariate Logistic Regression Analysis of Factors Associated With SWSD

Table 2 shows the final forward and backward stepwise multivariate logistic regression model with adjusted OR and 95% CI of the main variables. In terms of physical and psychological factors, compared to nurses who reported a low level of fatigue, those who reported a moderate or high level of fatigue were significantly more likely to experience SWSD (OR = 1.35, 95% CI: 1.08–1.67; and OR = 2.50, 95% CI: 2.02–3.10, respectively). A high perceived stress level (OR = 4.63, 95% CI: 2.60–8.63) had a statistically significant association with SWSD. Additionally, shift nurses who experienced physical discomfort during their night shift (OR = 2.05, 95% CI: 1.74–2.43) or experienced psychological stress before or after the night shift (OR = 1.51, 95% CI: 1.14–2.01; and OR = 1.52, 95% CI: 1.30–1.77, respectively) were more likely to develop SWSD than shift nurses who did not have such experiences.

**Table 2:** Stepwise multivariate logistic regression analysis of factors associated with shift work sleep disorder in shift nurses.

**Variables**	**Adjusted OR**	**95% CI**	***p* value**
**I. Physical and psychological factors**			
**Fatigue (ref: Low level)**			
Moderate	1.35	1.08–1.67	**0.007**
High	2.50	2.02–3.10	**<0.001**
**Physical discomfort during night shift (ref: No)**			
Yes	2.05	1.74–2.43	**<0.001**
**Psychological stress before night shift (ref: No)**			
Yes	1.51	1.14–2.01	**0.004**
**Psychological stress after night shift (ref: No)**			
Yes	1.52	1.30–1.77	**<0.001**
**Perceived stress (ref: Low level)**			
Moderate	1.23	0.97–1.57	0.094
High	4.63	2.60–8.63	**<0.001**
**II. Environmental and occupational factors**			
**Monthly income, Chinese Yuan (ref:** ** <3,000)**			
3,000–5,999	0.96	0.72–1.28	0.777
6,000–8,999	1.16	0.85–1.58	0.345
≥9,000	1.36	0.90–2.06	0.143
**Shift work experience (ref:** 0–5 years**)**			
6–10 years	0.93	0.75–1.15	0.506
11–15 years	1.26	0.98–1.61	0.072
16–20 years	1.42	1.00–2.02	0.051
>20 years	1.55	0.88–2.74	0.133
**Months of shift work per year (ref: 1–3 months)**			
4–6 months	1.18	0.78–1.81	0.435
7–9 months	1.55	1.03–2.35	**0.038**
10–12 months	1.79	1.26–2.58	**0.001**
**Night shifts per month (ref:** **≤4)**			
5–9	1.34	1.10–1.64	**0.005**
≥10	1.91	1.41–2.58	**<0.001**
**Days off after night shift (ref: 1 day)**			
1.5 days	0.76	0.61–0.95	**0.018**
≥2 days	0.77	0.65–0.91	**0.003**
**Interval between night shifts (ref:** **≤4 days)**			
5–7 days	0.93	0.79–1.09	0.359
≥8 days	0.69	0.52–0.92	**0.011**
**Night shift staffing (ref: 1)**			
2 (take turns)	0.70	0.58–0.84	**<0.001**
2 (take no turns)	0.96	0.76–1.21	0.736
≥3	1.05	0.83–1.31	0.704
**Busyness during night shift (ref: No)**			
Yes	1.30	1.09–1.55	**0.004**
**Working** **>40 h/week (ref: Never/rarely)**			
1 week/month	1.26	0.98–1.61	0.067
2 weeks/month	1.59	1.24–2.05	**<0.001**
3 weeks/month	1.95	1.47–2.60	**<0.001**
4 weeks/month	2.36	1.88–2.95	**<0.001**
**III. Social and behavioral factors**			
**Children (ref: 0)**			
1	1.14	0.92–1.40	0.226
≥2	0.88	0.70–1.10	0.244
**Work-family balance (ref: Low level)**			
High	0.41	0.29–0.56	**<0.001**
**Social support (ref: Low level)**			
Moderate	0.88	0.70–1.09	0.233
High	0.74	0.60–0.91	**0.005**
**Sleep duration before night shift (ref:** ** <3 h)**			
3–5 h	0.88	0.75–1.04	0.136
6–8 h	1.26	0.82–1.92	0.290
>8 h	1.95	1.08–3.55	**0.027**
**Naps per week (ref: Never/rarely)**			
≤ 1 day	0.83	0.67–1.03	0.090
2–3 days	0.77	0.63–0.94	**0.010**
4–5 days	0.93	0.71–1.21	0.582
6–7 days	0.75	0.51–1.08	0.123
**Using sleep medication before night shift (ref: Never/rarely)**			
Sometimes	1.45	1.14–1.85	**0.002**
Often	2.40	1.39–4.20	**0.002**
**Using sleep medication after night shift (ref: Never/rarely)**			
Sometimes	1.52	1.17–1.97	**0.002**
Often	3.25	1.86–5.89	**<0.001**
**Food intake during shift work (ref: Normal)**			
More	1.73	1.32–2.26	**<0.001**
Less	1.15	0.95–1.39	0.144
**Time of meal during shift work (ref: Regular)**			
Early	1.34	1.07–1.70	**0.012**
Delayed	1.39	1.15–1.68	**0.001**
**Physical activity (ref: Low level)**			
Moderate	1.14	0.93–1.40	0.202
High	1.39	1.17–1.64	**<0.001**

*CI, confidence interval; OR, odds ratio; ref, reference group*.

*Bold value for p < 0.05*.

In terms of environmental and occupational factors, nurses who worked shifts of 7–9 or 10–12 months per year were significantly more likely to experience SWSD than those who worked shifts of ≤ 3 months per year (OR = 1.55, 95% CI: 1.03–2.35; and OR = 1.79, 95% CI: 1.26–2.58, respectively). Furthermore, 5–9 and >10 night shifts per month showed a detrimental effect to the sleep of shift nurses, with ORs of 1.34 (95% CI: 1.10–1.64) and 1.91 (95% CI: 1.41–2.58), respectively. Shift nurses who worked more than 40 h per week were also more likely to develop SWSD than those who worked less (>40 h per week for more than 4 weeks/month: OR = 2.36, 95% CI: 1.88–2.95). Compared to nurses who rested for 1 day after each night shift, those who took 1.5 days and ≥2 days off after each night shift were significantly less likely to experience SWSD. (OR = 0.76, 95% CI: 0.61–0.95; and OR = 0.77, 95% CI: 0.65–0.91, respectively). Nurses with ≥8-day intervals between shifts were less likely to develop SWSD than those with ≤ 4-day intervals (OR = 0.69, 95% CI: 0.52–0.92). Busyness during night shift was also associated with SWSD (OR = 1.30, 95% CI: 1.09–1.55), and shift nurses are less likely to experience SWSD when two clinical nurses were on the night shift and took turns to rest (OR = 0.69, 95% CI: 0.52–0.92).

In terms of social and behavioral factors, nurses who had a good work-family balance (OR = 0.41, 95% CI: 0.29–0.56) or good social support (OR = 0.74, 95% CI: 0.60–0.91) were less likely to experience SWSD. Nurses who responded that they could get >8 h of sleep before night shift were statistically significantly more likely to have SWSD (OR = 1.95, 95% CI: 1.08–3.55) than those who got <3 h of sleep before night shift. In addition, napping 2–3 times per week was a protective factor for SWSD (OR = 0.77, 95% CI: 0.63–0.94). Shift nurses who often used sleep medication before or after night shifts were more likely to experience SWSD than those never used sleep medication (OR = 2.40, 95% CI: 1.39–4.20; and OR = 3.25, 95% CI: 1.86–5.89, respectively). Excessive eating and irregular eating were also main factors for SWSD. Finally, high-intensity physical activity was associated with increased odds of SWSD (OR = 1.39, 95% CI: 1.17–1.64).

### Feature Importance Ranking of the Factors Associated With SWSD on the Random Forest Model

**Figure 1:**
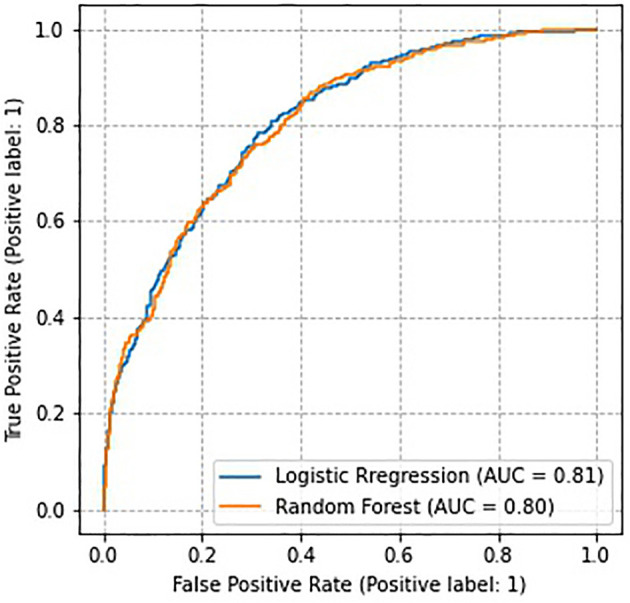
Receiver-operating characteristic curves for each predictive model.

Before analyzing the predictors, we evaluated the performance of the two models. The results showed that the predictive efficiency of logistic regression model (sensitivity = 0.73, specificity = 0.71, accuracy = 0.72, area under the receiver operating characteristic curve [AUROC] = 0.81) was similar to that of random forest model (sensitivity = 0.72, specificity = 0.71, accuracy = 0.72, AUROC = 0.80) (Figure 1). Therefore, the model prediction performance were guaranteed in our study.

**Figure 2:**
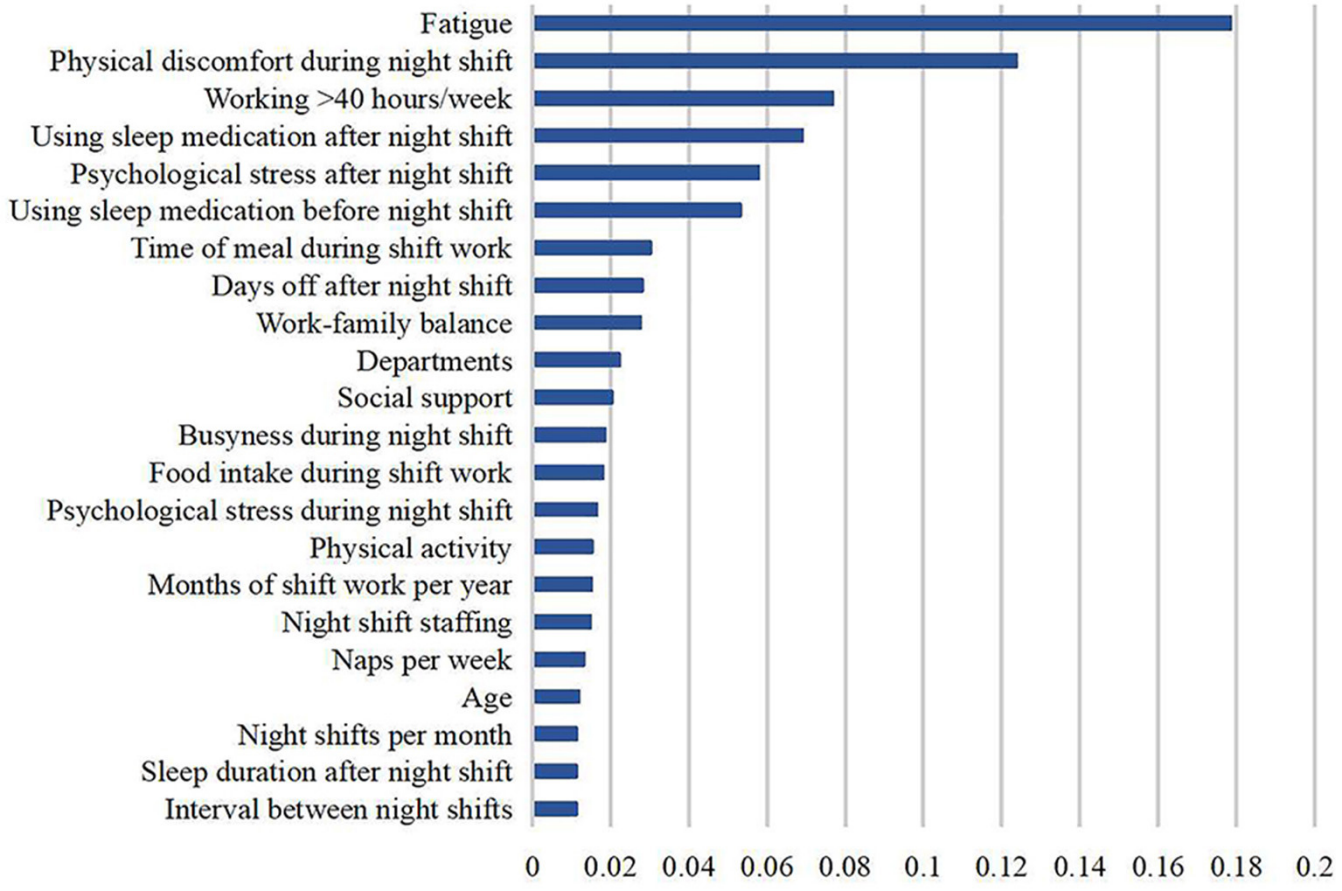
Feature importance ranking of the factors associated with shift work sleep disorder.

To illustrate the contribution of each factor to predictive capacity, we calculated the feature importance based on random forest. The feature importance of the top 50% of factors was visualized in Figure 2, including fatigue, physical discomfort, working overtime per week, using sleep medication, mental stress, diet, length of rest days, work-family balance, and departments. We found that the results of the two statistical methods were consistent, which can indirectly prove the robustness of the results of the logistic regression model.

## Discussion

### Prevalence of SWSD Among Nurses

The incidence of SWSD among participating nurses was 48.5%, which is higher than the rates of 37.6% (11) and 35.2% (4) reported in two studies in Norwegian nurses, and the rate of 24.4% reported by The Nurses' Sleep Health Project in Japan (10). Previous studies on the prevalence of SWSD in different populations have reported a prevalence of 23.3% in Norwegian oil rig shift workers (36), 19.9% in Chinese textile shift workers (37), 12.7% in Australian shift workers (38), and 8.1% in American shift workers from the community (39).

This discrepancy in the prevalence among the different studies could be attributable to the different shift schedules of the target populations, different national conditions or different times. Although China and Japan have similar nurse shift schedules, we collected data from 14 hospitals of different levels in areas of different economic levels, so as to more comprehensively reflect the prevalence of SWSD in nurses. Moreover, the incidence of SWSD may be higher since the time of our survey was the COVID-19 pandemic. Our result was somewhat supported by a systematic review stating that 36.36% of healthcare workers experienced work-related sleep problems during the COVID-19 pandemic (40). In addition, differences in disease definitions or questionnaire content might also reflect this divergence. In 2009, some scholars developed a self-administered questionnaire based on the ICSD-2 (36). In 2014, ICSD-3 updated the criteria for SWSD, and Waage et al. updated the questionnaire content according to the updated criteria (4). In ICSD-2, SWSD is defined as insomnia and/or excessive sleepiness that overlaps with shift work hours and cannot be explained by other diseases (7). In ICSD-3, the criteria for SWSD included a reduction in total sleep time as a symptom and increased the minimum duration of symptoms associated with shift work scheduling from 1 to 3 months (8). ICSD-3 was more rigorous in terms of duration and total sleep time was added as an indicator to make the diagnosis more sensitive, leading to significant differences between the two criteria in estimating the incidence of SWSD. Unlike most previous studies based on ICSD-2 (10, 11, 36), we used the latest questionnaire based on the ICSD-3 criteria to obtain a more rigorous and sensitive diagnosis of SWSD.

### Physical and Psychological Factors

Similar to the results of previous studies, characteristics of physical and psychological factors showed a statistically significant relationship with the prevalence of SWSD among nurses; however, there were some differences in specific variables. For instance, SWSD is essentially a circadian rhythm disorder that has been associated with polymorphisms in various molecular clock genes (41). Languidity and flexibility are two characteristics of the circadian rhythm, and several previous studies have shown that fatigue may influence SWSD, while flexibility is a protective factor (19, 42, 43). Contrary to our hypothesis, we found no relationship between SWSD and circadian rhythm characteristics. One possible reason for this unexpected finding could be that approximately 70% of the circadian rhythms of shift nurses measured by the questionnaire in this study were at a moderate level, suggesting that a more objective and sensitive measurement method could help explore the relationship between SWSD and circadian rhythm characteristics in greater detail. Furthermore, in our study, nurses who reported experiencing fatigue and physical discomfort (e.g., headache, heart discomfort, gastrointestinal symptoms) during their night shifts were more likely to develop SWSD than those who did not, supporting the results reported previously (15, 23, 42, 44). Interestingly, high work stress during shift work and difficulty in falling asleep were previously found to be the typical symptoms of SWSD (45), consistent with our findings. In addition, the sensitivity of sleep to stress is a known predictor of SWSD (19, 46, 47), suggesting that reducing the mental burden of nursing work has potential benefits in reducing the incidence of SWSD. In addition, Afshari et al. reported that nurses have low levels of psychological resilience during the COVID-19 pandemic (48). Therefore, it is necessary to improve psychological resilience to relieve stress levels and indirectly reduce the impact of stress on SWSD.

### Environmental and Occupational Factors

Haile et al. proposed that three shifts are statistically significantly related to SWSD (49), which is different from our finding. We found that nurses on two shifts had a higher prevalence of SWSD than nurses on three shifts, although the results of the stepwise multivariate logistic regression analysis showed no statistically significant correlation between SWSD and rotation schedule, which supported the results of Vanttola et al. (19). Thus, we will continue to investigate the effect of shift schedule on SWSD with prospective follow-up data to verify the current results. An interesting finding of our study was that the number of shift years had no effect on SWSD, but more than 6 months of shift scheduling per year was associated with a higher likelihood of having SWSD. In addition, SWSD was correlated with the average number of nights per month, and more than four night shifts were detrimental to nurses' sleep, which is similar to that reported in other studies (21, 49). This suggests that in nursing management, the head nurse should schedule no more than four night shifts per month to prevent SWSD among nursing staff. Short rest periods (<11 h) between work shifts have been shown to increase the likelihood of SWSD in nurses (44, 50). This supports our finding that more than 1 day of rest (>12 h) after a night shift was a protective factor for SWSD in our study. Moreover, our study reported a novel finding that a busy night shift with one nurse can easily lead to SWSD, but a night shift where two nurses take turns to rest reduces the odds of SWSD. An interval of 8 days or more between night shifts helps nurses adapt to shifts and reduces the odds of SWSD. Working overtime (>40 h/week) for more than a week per month affected sleep. Di Milia et al. also found a statistically significant association between SWSD and weekly work hours (43).

### Social and Behavioral Factors

In our study, the marital status of nurses and whether or not they had children had no association with SWSD, but a higher work-family balance or social support (i.e., family support and friend support) was associated with a lower likelihood of SWSD. Among nurses, the level of social support was statistically significantly and negatively associated with insomnia and daytime sleepiness (51), which supported our conclusions. Our results differed from those of previous studies (49), which only took into account marital status and children, possibly ignoring the more important variable of work-family balance. Afshari et al. reported that nurses have increased demands for social support and work-family balance in the context of the COVID-19 pandemic (48). Therefore, future research can focus more on the influence of psychological factors related to family and society on SWSD.

Numerous studies have repeatedly identified the use of multiple hypnotic agents (such as exogenous melatonin and injectable sleep medications) as predictors of SWSD (21, 49, 52), which is consistent with the findings of our research. We also investigated the role of sleep time before and after the night shift, rest taken during the night shift, and weekly naps. Those with SWSD had greater sleep debt on shift days and slept less compensatory sleep on days off than those without SWSD (19). Hence, our results suggest that effective sleep supplementation should include sleeping for <8 h before the night shift, with 2–3 compensatory naps per week. It is vital to raise awareness and education on sleep hygiene practices among nurses (53). We innovatively explored the effects of diet on SWSD during shifts. In our study, we found that irregular eating during the shift was a predictor of SWSD, but the results should be interpreted with caution and studies should be carried out in the future to verify our findings. Our results show that high-intensity physical activity was positively correlated with SWSD. However, a previous study contradicted our findings (54). One possible reason for the contradictory results is that we used a self-administered questionnaire to assess physical activity whereas Hajo et al. used objective ActiGraph GT3X accelerometers for data collection (54, 55). Therefore, future studies can combine self-reporting and objective monitoring to further explore the effect of physical activity on SWSD.

### Limitations

One limitation is the cross-sectional nature of this analysis. Further studies should collect longitudinal data to better understand the causal relationship between factors and SWSD. In addition, this study only included hospitals in Shandong Province in China; therefore, caution should be exercised when generalizing the study's findings to other regions. Our study lacks objective indicators; thus, future studies with objective measurements will help to verify the accuracy of the results of the current study.

## Conclusion

The prevalence of SWSD in the sampled shift nurses was 48.5% during the COVID-19 pandemic. To reduce the incidence of SWSD for nurses, head nurses and health care organizations need to develop more rational and scientific scheduling strategies and health education for nurses on the basis of the Integrated Framework for Population Health Risk Management. For example, fatigue, perceived stress, and body discomfort were among the physical and psychological factors associated with SWSD. Therefore, nursing managers should pay attention to the physical and mental health of nurses and offer timely interventions. In terms of environment and occupation, good scheduling strategies included placing two nurses on night shifts and allowing them to take turns to rest, arranging for shift nurses to rest for more than 1 day after night shifts, and controlling shift intervals for more than 8 days. However, arranging nurses in continuous shifts for more than 6 months, scheduling more than four night shifts per week, and working overtime were all bad scheduling strategies. Positive social and behavioral factors included 6–8 h of supplementary sleep after the night shift, 2–3 naps per week, eating regularly and ensuring adequate water intake per day, and improving family and social support. Therefore, health education for nurses should include good sleep strategies and maintenance of good family and social relations.

## Data Availability Statement

The datasets presented in this article are not readily available because the data from the Nurses' Health Cohort Study of Shandong needs time for data clearing and establishment of guidelines. We are planning on opening this data to the public in the future. Requests to access the datasets should be directed to caoyj@sdu.edu.cn.

## Ethics Statement

The studies involving human participants were reviewed and approved by the Ethics Committee of Scientific Research of Shandong University Qilu Hospital. The patients/participants provided their written informed consent to participate in this study.

## Author Contributions

YL and YW: methodology, formal analysis, data curation, software, writing-original draft, and visualization. RL and XL: writing-review and editing and project administration. XG, LL, and JL: investigation. FX, XJ, and YC: conceptualization, resources, supervision, project administration, and funding acquisition. All authors contributed to the article and approved the submitted version.

## Funding

The study was funded by the National Key Research and Development Program of China (Grant number 2020YFC2003500). The funders had no role in study design, data collection and analysis, decision to publish, or preparation of the manuscript.

## Conflict of Interest

The authors declare that the research was conducted in the absence of any commercial or financial relationships that could be construed as a potential conflict of interest.

## Publisher's Note

All claims expressed in this article are solely those of the authors and do not necessarily represent those of their affiliated organizations, or those of the publisher, the editors and the reviewers. Any product that may be evaluated in this article, or claim that may be made by its manufacturer, is not guaranteed or endorsed by the publisher.
